# Anti-Inflammatory Effect of Resiniferatoxin During Gingival Tissue Inflammation After Mechanical Pulp Damage in a Murine Experimental Model

**DOI:** 10.3390/ijms27052143

**Published:** 2026-02-25

**Authors:** José Luis Muñoz-Carrillo, Oscar Gutiérrez-Coronado, Gloria Stephanie Cortés-Cordero, Paola Trinidad Villalobos-Gutiérrez, Francisca Chávez-Ruvalcaba, María Isabel Chávez-Ruvalcaba, Maria Argelia Lopez-Luna, Oriana Rivera-Lozada, Joshuan J. Barboza

**Affiliations:** 1 Laboratorio de Inmunología, Centro Universitario de Los Lagos, Universidad de Guadalajara, Lagos de Moreno 47463, Jalisco, Mexico; ogutierrez@culagos.udg.mx (O.G.-C.); paola.villalobos2452@academicos.udg.mx (P.T.V.-G.); 2 Médica Morelos, Aguascalientes 20264, Aguascalientes, Mexico; stephycortes.sc@gmail.com; 3 Laboratorio de Toxicología y Farmacia, Área de Ciencias de la Salud, Universidad Autónoma de Zacatecas, Zacatecas 98160, Zacatecas, Mexico; charuva@uaz.edu.mx (F.C.-R.); iasruv9si@uaz.edu.mx (M.I.C.-R.); mariaa.lopez@uaz.edu.mx (M.A.L.-L.); 4 Vicerrectorado de Investigación, Universidad Señor de Sipán, Chiclayo 14002, Peru; riveraoriana@uss.edu.pe

**Keywords:** dexamethasone, gingival inflammation, ibuprofen, infiltrated inflammatory cells, prostaglandin-E_2_, pulp inflammation, resiniferatoxin, tumour necrosis factor-α

## Abstract

Gingival inflammation represents one of the most prevalent oral inflammatory conditions worldwide and remains a major contributor to oral morbidity. While its classical etiologies are well established, less attention has been paid to inflammatory responses that arise secondary to pulpal injury and tissue damage. Experimental models that allow controlled evaluation of these responses may provide relevant insight into pulp-associated gingival inflammatory processes. Current pharmacological approaches for inflammatory conditions in dentistry, including non-steroidal anti-inflammatory drugs (NSAIDs) and glucocorticoids, are widely used and generally effective. However, their use may be associated with adverse effects in specific clinical contexts, particularly under prolonged or high-dose regimens, highlighting the importance of continued investigation of additional pharmacological strategies. Within this context, pharmacological modulation of inflammatory pathways represents a relevant strategy for exploring alternative therapeutic approaches in pulp-associated gingival inflammation. Accordingly, there is a need to investigate novel molecules with therapeutic potential, such as resiniferatoxin, which has demonstrated anti-inflammatory properties in both in vitro and in vivo experimental models. This study aims to evaluate the anti-inflammatory effect of resiniferatoxin during inflammation of gingival tissue after mechanical pulp damage in a murine experimental model. Six groups of six BALB/c mice were formed as follows: five control groups: a healthy group (H_CG_), a healthy group treated with resiniferatoxin (RTX_HG_), a group with pulp damage at 14 h (PG_I_), two groups with pulp damage treated with ibuprofen (PGI_IBU_), dexamethasone (PGI_DEX_) at 14 h, and an experimental group with pulp damage treated with resiniferatoxin (PGI_RTX_) at 14 h. Gingival inflammation was evaluated after pulp damage was induced through mechanical pulp exposure of the upper first molar. The histopathological parameters of the gingival tissue of all groups were evaluated by hematoxylin and eosin (H&E) staining, while the plasma levels of PGE_2_ and TNF-α were quantified by ELISA assay. A significant increase in plasma PGE_2_ and TNF-α levels was observed at 14 h after pulp damage. Subsequently, when treatment with resiniferatoxin was administered, it was observed that this significantly decreased (* *p* < 0.05) the plasmatic levels of PGE_2_ and TNF-α, as well as the number of inflammatory cells infiltrated in the gingival tissue 14 h after the pulp damage, similar to treatments with ibuprofen and dexamethasone. Resiniferatoxin exerts an anti-inflammatory effect after pulp damage, significantly decreasing plasma levels of PGE_2_ and TNF-α, as well as the number of inflammatory cells infiltrated in the gingival tissue, which places resiniferatoxin as a potential drug, in this case, for the treatment of gingival inflammation.

## 1. Introduction

Pulp exposure and injury is a common phenomenon, caused by various stimuli, such as thermal, chemical, mechanical and microbiological [1], which are capable of inducing an inflammatory response in the pulp tissue [2]. Pulp inflammation is a protective physiological response that seeks to eliminate these harmful stimuli, thus initiating the healing/regeneration process of pulp tissue [3]. This process is mainly characterized by vasodilation and the recruitment and accumulation of inflammatory cells in the pulp tissue [4]. In addition, it also involves the production and release of several inflammatory mediators that influence the regeneration and repair of the dentin-pulp complex [5]. This pulp damage can potentially communicate with the periodontium, producing periodontal lesions and leading to the destruction of a large part of the alveolar bone [6,7].

Endo-periodontal lesions are clinical situations in which the periodontium and root canal of the same tooth become infected, leading to the destruction of the dental attachment apparatus [8], and can be interdependent due to the vascular and anatomical connections between the pulp and the periodontium [9]. Several etiological factors, such as bacteria, as well as contributing factors, such as trauma, root resorption, perforations, cracks and dental malformations, play an important role in the development and progression of endo-periodontal lesions [10]. Endo-periodontal lesions are caused by polymicrobial infections that can originate in the periodontium, pulp tissues, or both, activating an inflammatory response, which is mainly characterized by the activation of a wide variety of cells and the production of pro-inflammatory mediators, such as TNF-α and PGE_2_, which are involved with the severe development of these pathologies, through the destruction of tissues and the local resorption of the alveolar bone that supports the tooth [11,12,13]. The treatment of endo-periodontal lesions is complex and involves strict control of infection in both periodontal and pulp tissues [14]. This treatment varies depending on the etiology, pathogenesis and correct diagnosis of each specific pathology [10], which consists of root canal treatment when the origin is endodontic and non-surgical and/or a surgical periodontal therapy when the origin is periodontal [15,16,17]. On the other hand, during endo-periodontal lesions, pharmacological therapy for infection control consists of the use of antimicrobials [18,19,20] and antibiotics [21,22], while the pharmacological treatment of inflammation includes non-steroidal anti-inflammatory drugs (NSAIDs) [23,24] and, in some cases, glucocorticoids [25,26]. However, frequent or long-term use of these anti-inflammatory drugs is therapeutically limited due to their side effects [27,28,29]. In this context, there is still a need to develop new alternative therapies that contribute to the treatment of inflammatory diseases. Therefore, there is a wide variety of anti-inflammatory agents available and in development [30], which include biological agents, such as resiniferatoxin, which has demonstrated therapeutic potential for the treatment of inflammation.

Resiniferatoxin is a vanilloid derivative from the cactus-like plant *Euphorbia resinifera* [31], which is an ultra-potent agonist of the transient receptor potential vanilloid (TRPV)-1, with a unique spectrum of pharmacological action, since its therapeutic window is wide, allowing complete desensitization and inactivation of TRPV-1, decreasing the perception of pain and neurogenic inflammation [32]. In this context, diverse studies have reported that resiniferatoxin exhibits anti-inflammatory properties in both in vitro and in vivo models [29,33]. In the field of dentistry, a recent study evaluated the anti-inflammatory effect of resiniferatoxin in an experimental model of pulp inflammation in mice, in which it was observed that resiniferatoxin was capable of significantly decreasing plasma levels of pro-inflammatory mediators, as well as the number of inflammatory cells infiltrated in the pulp tissue [34]. Based on this rationale, we hypothesized that systemic administration of resiniferatoxin would attenuate gingival inflammatory cell infiltration and reduce plasma levels of PGE_2_ and TNF-α following mechanical pulp injury in a murine experimental model. Given these findings, and the need to search for new therapeutic alternatives for the treatment of inflammatory diseases in the field of dentistry, the aim of this study was to evaluate the anti-inflammatory effect of resiniferatoxin during inflammation of gingival tissue after mechanical pulp damage in a murine experimental model.

## 2. Results

### 2.1. Plasma Levels of PGE_2_, TNF-α, and Histopathology in Gingival Tissue: 14 Hours After Pulp Damage

**Figure 1:**
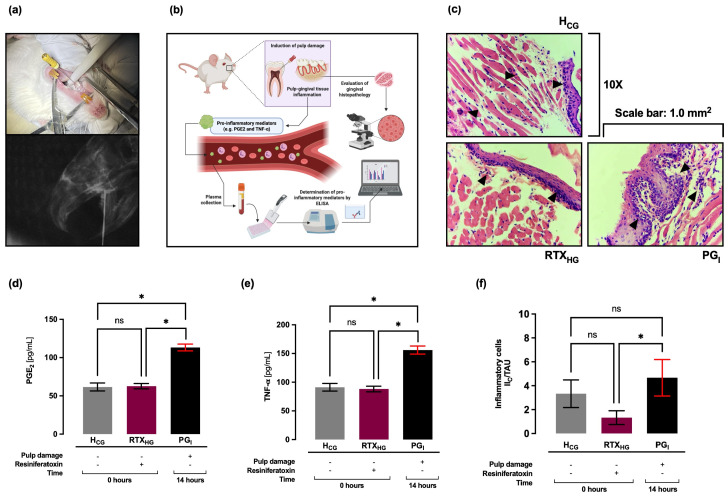
Induction of pulp damage (**a**) and illustration of the methodological summary (**b**). Histopathology of gingival tissue at 14 h after pulp damage (**c**). Representative histological images are shown; quantitative analysis focused on inflammatory cell infiltration within the connective tissue. Plasma levels of PGE_2_ (**d**) and TNF–α (**e**) at 14 h after pulp damage. Number of infiltrating inflammatory cells in gingival tissue at 14 h after pulp damage (**f**). Black arrows indicate infiltrating inflammatory cells. Values are represented as mean ± SD per group, indicating the level of significance (* *p* < 0.05). ns: not significant. H_CG_: healthy groups. RTX_HG_: healthy groups treated with resiniferatoxin. PG_I_: groups with pulp damage at 14 h.

Plasma levels of PGE_2_ and TNF-α were quantitatively determined by ELISA immunoassay, and histopathology of gingival tissue was evaluated 14 h after pulp damage in the control groups. It was observed that at 14 h (PG_I_ group) after pulp damage, plasma levels of PGE_2_ (113 ± 5 pg/mL) increased significantly (* *p* < 0.05) compared to the H_CG_ group (62 ± 5 pg/mL), while when compared to the RTX_HG_ group, a significant increase (* *p* < 0.05) in plasma levels of PGE_2_ (63 ± 4.0 pg/mL) was observed (Figure 1d). Regarding TNF-α, it was observed that at 14 h (PG_I_ group) after pulp damage, plasma levels (156 ± 7 pg/mL) increased significantly (* *p* < 0.05) compared to the H_CG_ (91 ± 7 pg/mL) and RTX_HG_ (88 ± 5.0 pg/mL) groups (Figure 1e). Furthermore, no statistically significant differences were found in plasma levels of TNF-α and PGE_2_ between the H_CG_ and RTX_HG_ groups.

Histologically, in both H_CG_ and RTX_HG_ groups, a basal level of infiltrated inflammatory cells (3 ± 1.0 II_C_/TAU and 1 ± 0.6 II_C_/TAU, respectively) was observed, without the presence of edema. Histological sections showed perigingivial soft tissues (skeletal muscle, mature adipose tissue, gingival mucosa and adnexa) without alterations, while in the PG_I_ group it was observed that, at the level of the subepithelial lamina propria, there was the presence of chronic inflammatory infiltrate (5 ± 2.0 II_C_/TAU, * *p* < 0.05), with mild and focal lympho-plasmacytic predominance (Figure 1c,f). No histological features of coagulative necrosis or extensive tissue destruction were identified in the gingival specimens.

### 2.2. Effect of the Treatment with Ibuprofen, Dexamethasone and Resiniferatoxin on the Plasma Levels of PGE_2_, TNF-α, and Histopathology in Gingival Tissue: 14 Hours After Pulp Damage

**Figure 2:**
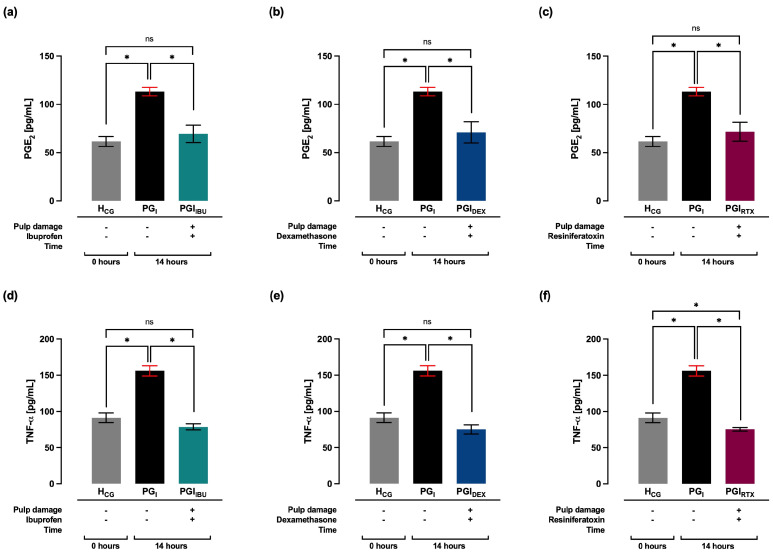
Plasma levels of PGE_2_ at 14 h after pulp damage of the groups treated with (**a**) ibuprofen, (**b**) dexamethasone and (**c**) resiniferatoxin. Plasma levels of TNF–α at 14 h after pulp damage of the groups treated with (**d**) ibuprofen, (**e**) dexamethasone and (**f**) resiniferatoxin. Values are represented as mean ± SD per group, indicating the level of significance (* *p* < 0.05). ns: not significant. H_CG_: healthy groups. PG_I_: groups with pulp damage at 14 h. PGI_IBU_: groups with pulp damage treated with ibuprofen at 14 h. PGI_DEX_: groups with pulp damage treated with dexamethasone at 14 h. PGI_RTX_: experimental groups with pulp damage treated with resiniferatoxin at 14 h.

At 14 h after pulp damage, PGE_2_ levels were observed to decrease significantly (* *p* < 0.05) in the groups treated with ibuprofen (PGI_IBU_, 69 ± 9 pg/mL), dexamethasone (PGI_DEX_, 71 ± 11 pg/mL) and resiniferatoxin (PGI_RTX_, 72 ± 10 pg/mL), compared to the PG_I_ group. Compared with the H_CG_ group, all three treatments showed similar plasma levels of PGE_2_ (Figure 2a–c). Similarly, it was observed that at 14 h after pulp damage, treatments with ibuprofen (PGI_IBU_, 79 ± 4 pg/mL), dexamethasone (PGI_DEX_, 75 ± 6 pg/mL) and resiniferatoxin (PGI_RTX_, 75 ± 3 pg/mL) significantly decreased (* *p* < 0.05) plasma levels of TNF-α, compared to the PG_I_ group. Compared with the H_CG_ group, ibuprofen and dexamethasone treatments showed similar plasma levels of TNF-α, except in the group treated with resiniferatoxin, which showed plasma levels of TNF-α lower (* *p* < 0.05) than the plasma levels of TNF-α of the H_CG_ group (Figure 2d–f).

**Figure 3:**
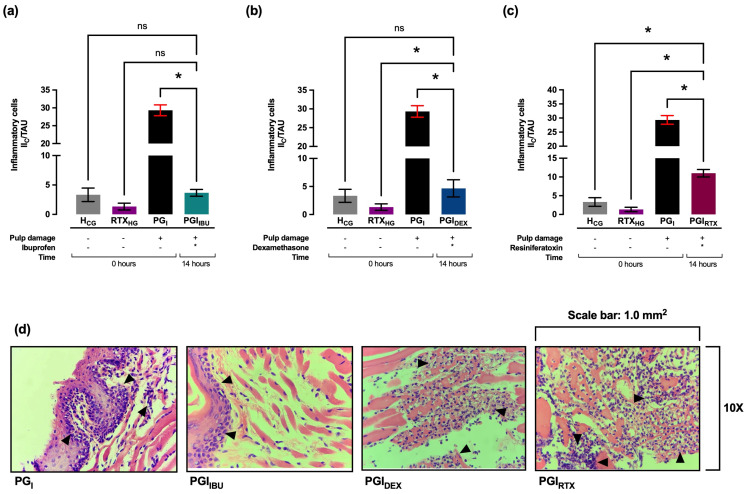
Number of infiltrated inflammatory cells during gingival tissue inflammation at 14 h after pulp damage in the groups treated with (**a**) ibuprofen, (**b**) dexamethasone, and (**c**) resiniferatoxin. (**d**) Representative histopathological images during gingival tissue inflammation at 14 h after pulp damage, in the groups treated with ibuprofen, dexamethasone, and resiniferatoxin. Variation in tissue orientation may result in differential visualization of epithelial and connective tissue layers across fields. Quantitative analysis was performed on standardized fields irrespective of epithelial representation, focusing exclusively on inflammatory cell infiltration within the connective tissue. Black arrows indicate infiltrating inflammatory cells. Values are represented as mean ± SD per group, indicating the level of significance (* *p* < 0.05). ns: not significant. H_CG_: healthy groups. RTX_HG_: healthy groups treated with resiniferatoxin. PG_I_: groups with pulp damage at 14 h. PGI_IBU_: groups with pulp damage treated with ibuprofen at 14 h. PGI_DEX_: groups with pulp damage treated with dexamethasone at 14 h. PGI_RTX_: experimental groups with pulp damage treated with resiniferatoxin at 14 h.

Histologically, it was observed that in treatments with ibuprofen (PGI_IBU_, 3 ± 1 II_C_/TAU), dexamethasone (PGI_DEX_, 4 ± 1 II_C_/TAU) and resiniferatoxin (PGI_RTX_, 4 ± 1 II_C_/TAU), the infiltrate of inflammatory cells decreased significantly (* *p* < 0.05) compared to PG_I_ group, presenting a mild focal inflammatory process. (Figure 3a–c). Furthermore, it was observed that in the PGI_IBU_ and PGI_DEX_ groups the inflammatory infiltrate was restricted to the subepithelial level, while in the PGI_RTX_ group the inflammatory infiltrate was located at the level of the musculoskeletal tissue (Figure 3d), without histological evidence of coagulative necrosis.

**Figure 4:**
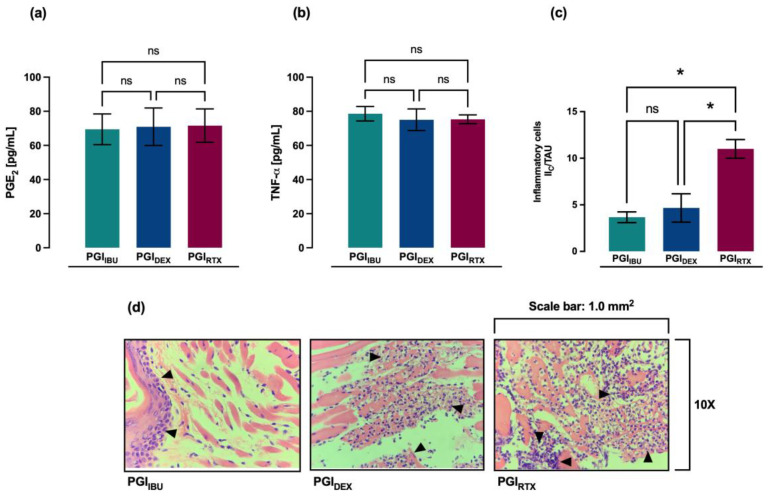
Comparison of the anti-inflammatory effect of treatments with ibuprofen (**a**), dexamethasone (**b**) and resiniferatoxin (**c**) on plasma levels of PGE_2_ and TNF-α at 14 h after pulp damage. (**d**) Representative histopathological images during gingival tissue inflammation at 14 h after pulp damage, in the groups treated with ibuprofen, dexamethasone, and resiniferatoxin. Variation in tissue orientation may result in differential visualization of epithelial and connective tissue layers across fields. Quantitative analysis was performed on standardized fields irrespective of epithelial representation, focusing exclusively on inflammatory cell infiltration within the connective tissue. Black arrows indicate infiltrating inflammatory cells. Values are represented as mean ± SD per group, indicating the level of significance (* *p* < 0.05). ns: not significant. PGI_IBU_: groups with pulp damage treated with ibuprofen at 14 h. PGI_DEX_: groups with pulp damage treated with dexamethasone at 14 h. PGI_RTX_: experimental groups with pulp damage treated with resiniferatoxin at 14 h.

Finally, the anti-inflammatory effect of the three treatments, ibuprofen (PGI_IBU_), dexamethasone (PGI_DEX_), and resiniferatoxin (PGI_RTX_), was compared. Regarding the level of proinflammatory cytokines in plasma, it was observed that there were no statistically significant differences in the plasma levels of PGE_2_ and TNF-α between the groups treated with ibuprofen (PGI_IBU_), dexamethasone (PGI_DEX_), and resiniferatoxin (PGI_RTX_), showing a similar anti-inflammatory effect, 14 h after pulp damage (Figure 4a–c). At the histological level, 14 h after pulp damage, a greater anti-inflammatory effect of treatments with ibuprofen (PGI_IBU_) and dexamethasone (PGI_DEX_) was observed, since both treatments significantly reduced the inflammatory infiltrate in the gingival tissue, compared to treatment with resiniferatoxin (PGI_RTX_). However, no significant difference in inflammatory infiltrate was observed between ibuprofen (PGI_IBU_) and dexamethasone (PGI_DEX_) treatments (Figure 4d).

## 3. Discussion

The dental pulp and the periodontium are intrinsically related from their embryonic origin, and although both compartments usually present independent pathologies, there are connections that allow the passage of microorganisms between them. Thus, the disease of one tissue can affect the other and generate endo-periodontal lesions [35]. Diverse investigations have studied the relationship between the pulp and periodontal disease [36], demonstrating that there is a correlation between both diseases [37,38]. In this sense, endo-periodontal lesions correspond to clinical manifestations derived from the inflammatory and pathological interaction between the pulp and periodontal tissues, which occurs through communication structures such as the apical foramen, lateral canals, accessory canals and dentin tubules [39,40].

The pathogenesis of endodontic and periodontal lesions is determined by multiple factors, including the microbial burden, the diversity, and virulence of the pathogens, and the interactions they establish with each other [41]. In this context, inflammatory mediators present in pulp and periodontal tissues play a fundamental role in the immune response to the infectious environment. These mediators exert pleiotropic effects, including the activation of inflammatory leukocytes, modification of vascular permeability, and the induction of bone resorption processes [42,43,44,45]. In the present study, plasma levels of proinflammatory mediators (PGE_2_ and TNF-α) as well as histopathological parameters of gingival tissue were evaluated after pulp tissue damage in a murine experimental model treated with resiniferatoxin, which is discussed in depth and detail below.

Arachidonic acid (ARA) that is esterified at the inner surface of the cell membrane is hydrolyzed to its free form by phospholipase A2 (PLA2) [46], which can subsequently be metabolized by diverse enzymes, such as lipoxygenases (LOX), cytochrome P450 enzymes (CYP) and cyclooxygenases (COX), producing a wide variety of bioactive mediators [47]. There are two main isoforms of COX: the COX-1 isoform, which is constitutively expressed in most tissues, and the COX-2 isoform, which is induced during inflammatory processes triggered by various stimuli, such as cytokines and endotoxins [48]. In this sense, the production of PGE_2_, an endogenous lipid mediator of inflammation, is regulated by COX-2 [49]. PGE_2_ plays a key role in generating the inflammatory response, as its biosynthesis increases significantly in inflamed tissue and contributes to the development of the cardinal signs of acute inflammation, such as edema and redness, as well as fever and hyperalgesia [50].

Current scientific evidence suggests that PGE_2_ plays a key role in the pathogenesis of endo-periodontal lesions by modulating the local immune response and promoting tissue destruction. During pulp tissue inflammation, PGE_2_ has been reported to increase vascular permeability [51] and enhance the pain response [52]. In addition, PGE_2_ is capable of inhibiting the osteogenic differentiation of mesenchymal stem cells [53], but it also stimulates the expression of mineralization genes in undifferentiated pulp cells [12], promoting the formation of tertiary dentin [54]. Clinical studies have reported a significant increase in PGE_2_ in cases of reversible and irreversible pulpitis, compared to healthy pulp [55,56,57]. Similarly, preclinical studies (experimental models in rats) have reported a significant increase in PGE_2_ during pulp inflammation [58], showing positive immunoreactivity for PGE_2_ three days after pulp exposure [59]. Finally, in vitro assays with human pulp cells show that PGE_2_ levels increase 24 h after stimulation with IL-1β and TNF-α in a dose-dependent manner [60]. During inflammation of the gingival tissue, studies have reported an important role for PGE_2_, since oral pathogens and proinflammatory cytokines (such as TNF-α, IL-1α, IL-1β) induce the expression of COX-2 and, therefore, the production of PGE_2_ in gingival fibroblasts, amplifying local inflammation [61,62]. In this context, the overproduction of PGE_2_ in periodontal tissues promotes vasodilation, increases vascular permeability, and enhances pain perception, causing the clinical signs of inflammation [63,64,65]. In addition, the vasoactive effects of PGE_2_ are also enhanced by synergistic interactions with other inflammatory mediators such as bradykinin, the complement system, and histamine [66]. On the other hand, several studies have reported elevated levels of PGE_2_ in gingival crevicular fluid [67,68], saliva, inflamed periodontal tissues, periapical exudates, and active endodontic lesions, which have been positively correlated with bleeding on probing, attachment loss, tissue destruction, and bone loss, as well as with the clinical severity of the disease and acute symptoms in endo-periodontal lesions [11,63,64,65,69,70].

TNF-α is a cytokine with pleiotropic effects, produced by various types of immune cells, mainly T lymphocytes, activated macrophages, and natural killer cells. At the physiological level, TNF-α is an important component of the normal immune response, which is essential for the initiation and progression of the inflammatory response, through the stimulation of the recruitment and migration of immune cells to the site of inflammation, and promotion of the synthesis of other inflammatory molecules, such as interleukins and chemokines. However, overproduction or improper function of TNF-α can be harmful, causing chronic inflammation and tissue damage, leading to diverse diseases [71,72].

TNF-α plays a central role in the pathogenesis of endo-periodontal lesions [73,74], as it drives inflammation, immune cell recruitment, and bone resorption. In the case of pulp inflammation, TNF-α has been observed to play a protective role, because it promotes odontoblastic differentiation [75,76], inducing greater mineralization through an increase in proteins associated with the formation of reparative dentin [77]. Studies in human dental pulp have reported that gene expression (mRNA) [78,79,80] and protein synthesis [81,82,83] of TNF-α increase significantly in cases of reversible and irreversible pulpitis, compared to healthy pulps. Likewise, studies in experimental animal models have reported maximum TNF-α expression three hours after LPS stimulation in rats [84], and twelve hours after pulp damage in mice [85]. On the other hand, TNF-α plays a crucial and fundamental role in protecting against periodontal pathogens [86]. Studies in animal models suggest that TNF-α plays an important role in the pathogenesis of periodontal disease, in the transition from gingivitis to periodontitis [87,88]. Likewise, clinical studies have reported a significant increase in local levels of TNF-α in gingival tissue, gingival crevicular fluid, saliva and periapical tissues in patients with chronic and apical periodontitis, which correlates with disease severity and tissue deterioration [89,90,91,92]. This destruction of periodontal tissues by TNF-α is related to several of its actions [93]. On the one hand, TNF-α induces the recruitment of circulating leukocytes [94] and stimulates the production of other proinflammatory mediators, which sustains the inflammatory response, and promotes the release of tissue enzymes and matrix metalloproteinases (MMPs), thus reducing the reparative capacity of the periodontium [95,96,97,98] and amplifying bone resorption in endo-periodontal lesions [99,100]. On the other hand, TNF-α participates in the stimulation of the differentiation and activity of osteoclasts, osteoblasts and osteocytes, increasing the expression of the receptor activator of the NF-κB ligand (RANK) and macrophage colony-stimulating factor (M-CSF), which leads to greater bone resorption in inflamed periodontal and periapical tissues, enhancing bone loss during endo-periodontal lesions [101,102,103,104,105].

The scientific evidence mentioned above highlights the importance of the role of PGE_2_ and TNF-α during pulp and periodontal inflammation. Several experimental and clinical studies have reported that during periodontal diseases, plasma levels of both PGE_2_ [69,106,107] and TNF-α [108,109,110] increase significantly, indicating local and systemic inflammation. Regarding pulp inflammation, the scientific evidence (mentioned previously) available to date has only reported the detection of PGE_2_ and TNF-α at the pulp tissue level. In particular, an experimental study conducted in a rat model reported that TNF-α levels increased significantly at the pulp and systemic levels, ninety minutes after systemic exposure to lipopolysaccharide (LPS), but not due to pulp damage [111]. Similarly, there is currently no reported information on the detection of systemic levels of PGE_2_ and TNF-α in endo-periodontal lesions. In this context, the present study is relevant as it is the first to report the detection of systemic levels of PGE_2_ and TNF-α in an experimental model of gingival tissue inflammation in BALB/c mice, since it was observed that at 14 h after pulp damage, there was a significant increase (* *p* < 0.05) in plasma levels of TNF-α and an upward trend in plasma levels of PGE_2_ in the PG_I_ group, compared to the control groups H_CG_ and RTX_HG_. This finding is consistent with the literature, as a study in an experimental model of pulp inflammation in BALB/c mice reported that plasma levels of PGE_2_ and TNF-α increased significantly (* *p* < 0.05) at 14 h after pulp damage [34]. Together, these findings indicate that both PGE_2_ and TNF-α are key mediators of the pulp and gingival inflammatory response, participating in the amplification of inflammation. Importantly, systemic levels of PGE_2_ and TNF-α were not intended to serve as direct surrogates of local gingival inflammation, but rather as complementary indicators of overall inflammatory status in response to pharmacological intervention.

Currently, NSAIDs are widely used for the treatment of inflammation, pain and fever [112], through the blocking of COX isoenzymes (COX-1 and COX-2), inhibiting the production of prostaglandins (PG) and thromboxanes (Tx) [113]. Ibuprofen is one of the most prescribed and used NSAIDs [114] for its analgesic, anti-inflammatory and antipyretic properties [115,116], which has a wide therapeutic index, since at high doses (1800–2400 mg/day) it is used for the treatment of chronic inflammatory diseases, while at low doses (800–1200 mg/day) it is indicated for the relief of mild (dental) pain and inflammation [117].

Several experimental and clinical studies have demonstrated that ibuprofen significantly reduces PGE_2_ levels in inflamed oral tissues, both pulp and periodontal tissues (Table 1), primarily by inhibiting arachidonic acid metabolism and COX activity [118,119,120,121,122,123,124,125,126]. In animal models and patients with irreversible pulpitis, ibuprofen treatment was associated with a significant decrease in PGE_2_ in pulp tissues and pulp blood samples [118,121,122]. Similarly, in vitro studies, clinical trials, and animal models of periodontitis showed marked inhibition of PGE_2_ production in gingival tissues and gingival crevicular fluid, accompanied by a reduction in postoperative pain and inflammation [123,124,125,126]. In contrast, evidence regarding the effect of ibuprofen on TNF-α is limited and contradictory (Table 1). Although a significant increase in TNF-α has been described during endo-periodontal lesions, some studies did not observe a significant reduction after ibuprofen treatment [120,127,128]. However, other studies have reported a decrease in TNF-α in blood samples obtained from the pulp of patients with irreversible pulpitis and stimulated gingival epithelial cells [118], and in gingival epithelial cells stimulated with *P. gingivalis*-LPS [129]. Recently, a study showed that, in an experimental model of pulp inflammation, ibuprofen significantly decreased (* *p* < 0.05) plasma levels of PGE_2_ and TNF-α [34]. In accordance with these findings, the present study demonstrates that ibuprofen significantly reduces (*p* < 0.05) plasma levels of PGE_2_ and TNF-α during gingival inflammation 14 h after pulp damage, providing novel evidence in a murine model that integrates the pathophysiological context of endo-periodontal lesions. Furthermore, these effects could be related not only to COX inhibition, but also to the modulation of the NF-κB pathway and the production of pro-inflammatory cytokines, such as TNF-α, similar to steroid anti-inflammatory drugs such as glucocorticoids [130,131,132,133].

Glucocorticoids are potent steroidal anti-inflammatory drugs, widely used for the treatment of inflammatory diseases [134]. Their main mechanism of action is genomic, mediated by interaction with their nuclear receptor (GR) [135]. The binding of glucocorticoids with their receptor GR forms an active protein complex (GC-GR), which translocates to the cell nucleus and binds to promoter regions of target genes in DNA, called glucocorticoids response elements (GREs), regulating gene expression, either positively or negatively [136]. Positive regulation causes gene transcription and translation of anti-inflammatory proteins, while negative regulation occurs through the suppression of pro-inflammatory gene expression, inhibiting transcription factors such as NF-κB [29,137].

Dexamethasone is a synthetic glucocorticoid with potent anti-inflammatory and immunosuppressive properties, widely used to modulate the exacerbated inflammatory response [138]. In pulpal diseases, most studies have focused on its efficacy in controlling endodontic pain, particularly in inferior alveolar nerve blocks [139,140,141,142,143,144], with limited evidence on its direct effect on pulpal inflammation and PGE_2_ and TNF-α levels. However, recent studies have shown that dexamethasone, administered locally or systemically, reduces TNF-α expression, promotes dentin mineralization [145], and significantly decreases (* *p* < 0.05) plasma levels of PGE_2_ and TNF-α at the systemic level, following pulp damage in an experimental model of pulpal inflammation in mice [34] (Table 2). In the periodontal context, dexamethasone has shown preventive effects on postoperative pain [146] and marked anti-inflammatory activity in in vitro studies and animal models [147,148,149,150,151,152]. At the cellular level, dexamethasone inhibits the expression of microsomal prostaglandin E synthase-1 (mPGES-1) [153,154], COX-2 [155,156], as well as the production of PGE_2_ [157,158,159] and TNF-α [160,161] in human gingival fibroblasts (GHFs) and human periodontal ligament (PDL) cells [162], previously stimulated with pro-inflammatory mediators, such as IL-1β, TNF-α, bradykinin and LPS. In animal models, dexamethasone significantly inhibited the increase in TNF-α associated with periodontitis [163], and reduced periodontal inflammation and alveolar bone loss, although alterations in mineral density and increased bone porosity have also been described [164,165,166,167] (Table 2). In accordance with this evidence, the present study demonstrates for the first time that dexamethasone significantly reduces (* *p* < 0.05) systemic levels of PGE_2_ and TNF-α during gingival inflammation 14 h after the pulp damage in a murine model, providing novel evidence in the pathophysiological context of endo-periodontal lesions.

Regarding dexamethasone, its prolonged therapeutic use can suppress the local immune response, delay healing, and promote secondary infections [145,168]. Systemic treatment or high doses alter the function of pulp cells and reduce the number of osteoblasts and osteoclasts, affecting bone repair and mineral density, with an impact on tissue homeostasis [169,170]. Furthermore, chronic therapeutic use of dexamethasone can cause gingival ulceration, attachment loss, periodontal fiber alterations, and alveolar bone loss, due in part to the inhibition of fibroblast activity and collagen formation, which delays tissue repair and promotes local osteoporosis [170,171]. In addition, the immunosuppression associated with dexamethasone can exacerbate periodontitis and induce spontaneous alveolar bone loss, especially at high doses [166]. At a systemic level, prolonged treatment with dexamethasone has been associated with delayed wound healing, increased risk of infection, mild hyperglycemia, and osteoporosis [141,172,173,174,175,176,177].

Currently, there is a wide variety of anti-inflammatory agents available [30]. However, given the limited therapeutic use of ibuprofen and dexamethasone in pulpal and gingival inflammation, there is still a need to develop new therapeutic alternatives that contribute to the treatment of inflammation in dental practice, in particular, through the use of biological products, such as resiniferatoxin. In addition to resiniferatoxin acting as a molecular analgesic by desensitizing nerves that express TRPV1 [178], several studies have shown that resiniferatoxin has a significant anti-inflammatory activity or effect. In vitro studies have reported that resiniferatoxin was capable of inhibiting NF-κB expression in a dose-dependent manner in the human leukemic myelomonoblastic (ML-1a) cell line stimulated with TNF-α [179], as well as iNOS and COX-2 expression in RAW264.7 macrophages stimulated with LPS and IFN-γ, resulting in a decrease in PGE_2_ and NO [180]. On the other hand, in vivo studies in models of ischemic acute renal failure (ARF) [181], *Trichinella spiralis* infection [182,183,184,185], and LPS-induced inflammation have demonstrated the anti-inflammatory and immunomodulatory effects of resiniferatoxin, which significantly reduced levels of pro-inflammatory mediators such as TNF-α, NO, PGE_2_, IL-12, IFN-γ and IL-1β, while increasing anti-inflammatory cytokines such as IL-10, IL-4, and IL-13, possibly through the NF-κB signaling pathway, independent of TRPV-1 receptors [33]. Another study evaluated the effect of resiniferatoxin in a murine model with LPS-induced inflammation. It was observed that treatment with resiniferatoxin significantly reduced plasma levels of PGE_2_, NO, IL-1β and TNF-α. In addition, Bay 11-7082 and CPZ combined with resiniferatoxin showed a synergistic effect, decreasing inflammatory markers. These findings suggest that the anti-inflammatory effect of resiniferatoxin is apparently associated with the NF-κB signaling pathway, independent of TRPV-1 receptors [33]. However, this is the first report indicating that resiniferatoxin treatment decreases the systemic production of these proinflammatory mediators during gingival tissue inflammation after pulp damage in a murine experimental model.

Periodontal lesions are characterized by an increase in neutrophils, which, through the release of chemokines, promote the recruitment and activation of immune cells, as well as osteoclastic bone resorption, contributing to both the onset and progression of periodontitis [186]. In addition, macrophages represent a relevant source of pro-inflammatory mediators such as IL-1β, TNF-α, MMP and PGE_2_, whose elevated levels in gingival tissue and CGF are directly associated with the severity of periodontal disease [187]. Similarly, chronic pulp inflammation is characterized by inflammatory infiltrate, fibrosis, and progressive tissue deterioration, which can culminate in pulp necrosis and loss of defensive function [188]. A study of an experimental model of pulp inflammation in mice showed that 14 h after pulp damage, there was a mild infiltrate of inflammatory cells, presence of edema, vascular leakage, and necrosis of the pulp tissue, while 18 h after pulp damage, a significant increase in the infiltrate of inflammatory cells to moderate was observed, with the presence of edema, vascular leakage, and no pulp necrosis [34]. Histopathological evidence indicates that periodontitis can induce inflammation, fibrosis, and pulp necrosis, confirming the close interrelationship between gingival and pulp inflammation [189]. In our study, it was observed that, 14 h after pulp damage, there was a significant increase (* *p* < 0.05) in the chronic infiltrate of inflammatory cells. Furthermore, when treatments with IBU and dexamethasone were administered, it was observed that 14 h after pulpal damage, the inflammatory cell infiltrate decreased significantly, presenting a mild focal inflammatory process. Our results are consistent with what has been reported in the literature, as it was observed that when treatment with ibuprofen, dexamethasone and resiniferatoxin was administered, it significantly reduced the number of cells infiltrated in the pulp tissue, but with the presence of edema, vascular leakage and pulp necrosis [34]. This reduction in the observed inflammatory infiltrate is due to the fact that GCs such as dexamethasone are capable of inducing apoptosis in immune system cells [190,191,192], inhibiting chemotaxis [193] and cell proliferation [194]. In the case of ibuprofen, it has also been observed that it is capable of inhibiting cell proliferation [132]; however, further studies are needed to confirm this hypothesis. Although the inflammatory infiltrate likely comprises a mixture of mononuclear and polymorphonuclear cells, the histological assessment was limited to quantification of total inflammatory cell infiltration using hematoxylin and eosin staining, without cell-type-specific identification. Future studies incorporating cell-specific markers or immunohistochemical approaches, such as myeloperoxidase staining, may further refine characterization of inflammatory cell populations.

Regarding resiniferatoxin, studies in rat models with *T. spiralis* infection have reported that resiniferatoxin treatment significantly reduced plasma and intestinal eosinophils. [182,183,184,185]. Furthermore, when treatment with resiniferatoxin was administered, in a study of an experimental model of pulp inflammation in mice, it was observed that 14 h after pulpal damage, the inflammatory cell infiltrate significantly decreased necrosis [34]. These findings are consistent with our results, as this is the first study to show that resiniferatoxin treatment is capable of reducing the number of inflammatory cells infiltrated in the gingival tissue, 14 h after pulp damage. Although there is no clear evidence of the mechanisms by which resiniferatoxin presents this effect, our hypothesis is based on the fact that this effect of resiniferatoxin could be associated with its ability to negatively regulate the production of proinflammatory mediators, since the survival of inflammatory cells is associated with the production of proinflammatory cytokines [195,196]. However, more studies are needed to confirm this hypothesis. Accordingly, while pulp inflammation represents the initiating event, the present study specifically addresses the downstream gingival inflammatory response, providing novel evidence of the systemic and periodontal anti-inflammatory effects of resiniferatoxin. Although resiniferatoxin is known to interact with sensory neurons, the present study was not designed to investigate the neural mechanisms underlying its anti-inflammatory effects, which remain an important subject for future investigation.

Resiniferatoxin has become a potent analgesic and anti-inflammatory compound due to its high affinity for TRPV-1 receptors and its ability to induce long-lasting desensitization of nociceptive neurons [197]. In addition to its neuromodulatory effects, increasing evidence suggests that resiniferatoxin exerts immunomodulatory actions by inhibiting NF-κB signaling and suppressing the production of pro-inflammatory mediators, such as PGE_2_ and TNF-α. These mechanisms could explain the significant reduction in these inflammatory mediators at the systemic level and in the gingival inflammatory infiltrate observed in the present study. In this context, when comparing the anti-inflammatory effect of the three treatments with ibuprofen, dexamethasone, and resiniferatoxin in the present study, no statistically significant differences were observed in plasma levels of PGE_2_ and TNF-α, showing a similar anti-inflammatory effect 14 h after pulp damage. Histologically, 14 h after pulp damage, a greater anti-inflammatory effect was observed with the ibuprofen and dexamethasone treatments compared to the resiniferatoxin treatment; however, all three treatments showed a significant reduction in inflammatory infiltrate. Our findings are consistent with previous reports, as various experimental studies in animal models have shown that resiniferatoxin has a similar anti-inflammatory effect to treatment with other anti-inflammatory drugs such as ibuprofen and dexamethasone, at a lower therapeutic dose, demonstrating efficacy and pharmacological potency [33,34,183,184,185]. However, despite its promising anti-inflammatory properties, the therapeutic use of resiniferatoxin is not without potential limitations. At high doses or with inappropriate routes of administration [198], resiniferatoxin treatment has been reported to be associated with cytotoxic effects, neuronal degeneration, and prolonged sensory deficits due to excessive calcium influx mediated by TRPV-1 [199,200]. Furthermore, resiniferatoxin-induced neurotoxicity and localized tissue damage have been described in some experimental models [201,202,203,204]. High doses of resiniferatoxin have also been reported to cause severe inflammation of the digestive mucosa, gastroenteritis, abdominal pain, vomiting, hematuria, arrhythmia, pulsatile seizures, and death by asphyxiation [205], highlighting the importance of dose optimization, controlled delivery systems, and careful evaluation of safety profiles. Alternative routes of administration, including localized delivery, represent an important area for future investigation but were beyond the scope of the present systemic pharmacological evaluation. Therefore, while resiniferatoxin represents a promising alternative to conventional anti-inflammatory drugs, further preclinical studies are required to define its therapeutic window, long-term safety, and optimal administration strategies before its translation into dental clinical applications.

### Study Limitations and Future Directions

This study has several limitations that should be acknowledged. First, the experimental model was intentionally designed to evaluate pulp–gingival inflammation induced by controlled mechanical pulp exposure, allowing isolation of this specific stimulus. Accordingly, the findings should be interpreted within the context of a mechanical injury model rather than as a comprehensive representation of all etiological factors associated with endo-periodontal injuries. Other etiological stimuli, including thermal, microbiological, chemical, or caries-driven models, may activate distinct inflammatory pathways and warrant investigation in future studies.

Second, although pulpal damage served as the initiating stimulus, histopathological analyzes were focused on gingival tissue in accordance with the primary objective of the study. Pulpal inflammatory changes following mechanical injury and resiniferatoxin treatment have been previously characterized [34] and were therefore not reassessed. In addition, systemic inflammatory mediators were prioritized as outcome measures to capture the overall inflammatory response. Future studies incorporating simultaneous pulp and gingival tissue analysis, as well as tissue-specific transcriptional profiling, may provide further mechanistic insight.

The inflammatory response to pulp exposure is dynamic and evolves over time. In this study, a 14 h time point was selected to assess early systemic and gingival inflammatory responses based on prior evidence. While appropriate for evaluating acute pharmacological modulation, additional time points (24–72 h), as reported in other murine models [85], would be valuable to characterize temporal progression and longer-term effects.

No postoperative analgesia was administered to avoid pharmacological interference with inflammatory pathways. While this may have contributed to elevated baseline systemic inflammatory mediators, all experimental groups were exposed to identical nociceptive conditions, allowing relative comparisons between treatments to remain interpretable. It should be noted that anti-inflammatory treatments were administered after the inflammatory response was established; therefore, the present study evaluates the acute pharmacological modulation of an ongoing pulp–gingival inflammatory process rather than the prevention or early development of inflammation. Additionally, because treatments were administered systemically, changes in circulating inflammatory mediators may reflect effects beyond the gingival lesion itself, including systemic or off-target pharmacological actions.

Finally, although animals were homogeneous and standardized procedures, formal randomization, blinding, and a priori power calculations were not implemented. While the sample size (*n* = 6 per group) was sufficient to detect statistically significant differences in this exploratory study, these methodological refinements should be incorporated in future confirmatory investigations to further strengthen rigor and reduce the risk of type II errors.

## 4. Materials and Methods

### 4.1. Experimental Animal Model

Male BALB/c mice of two and a half months of age, with an average body weight of 25 g, were used. Six groups of six mice each were formed: a healthy control group (H_CG_); a healthy group treated with resiniferatoxin (RTX_HG_); a control group with pulp–gingival inflammation at 14 h (PG_I_); a pharmacological comparator group with pulp–gingival inflammation and treated with ibuprofen at 14 h (PGI_IBU_); a pharmacological comparator group with pulp–gingival inflammation and treated with dexamethasone at 14 h (PGI_DEX_); and an experimental group with pulp–gingival inflammation and treated with resiniferatoxin at 14 h (PGI_RTX_). Animals were assigned to experimental groups based on predefined experimental design (animals used were homogeneous in terms of strain, sex, age, and weight); however, no formal randomization or blinding procedures were applied. This study was reviewed and approved by the Bioethics Committee of the Health Sciences Area of the Autonomous University of Zacatecas, with the bioethical approval number ACS/UAZ/083/2019 and was made in accordance with the Official Mexican Norm (NOM-062-ZOO-1999), published by the Secretariat of Agriculture, Livestock, Rural Development, Fisheries and Food (SAGARPA) in the Official Gazette of the Federation (México) on 28 June 2001.

### 4.2. Induction of Pulp Lesion

Pulp lesion was performed according to previously published methods (Figure 1a,b, in Section 2 of results) [34,85]. The dental cavities of the PG_I_, PGI_IBU_, PGI_DEX_, and PGI_RTX_ groups were prepared on the occlusal surface of the bilateral upper first molars (class 1 cavity), with a #1/4 dental round bur, under a surgical microscope (40×). The upper first molars were drilled using a dental micromotor at approximately 20,000–30,000 rpm with a cooling system, until the pulp was observed through the transparency of the dentin floor of the cavity. Pulp exposure was performed using an endodontic hand file (0.15 mm diameter tip, 2% taper), gently inserted approximately 1–1.5 mm into the pulp chamber, performing 2–3 controlled strokes over a period of 5–10 s, avoiding excessive pressure to standardize the extent of mechanical damage, leaving the cavity open and exposed to the oral environment. Subsequently, it was determined whether the pulp lesion induced damage (inflammation) in gingival tissue (see gingival histopathology).

### 4.3. Drug Treatment

#### 4.3.1. Anesthetic Treatment

All mice were anesthetized with sodium pentobarbital (PISABENTAL^®^, Reg. SAGARPA Q-7833-215) with a dose of 50 mg/Kg, administered intraperitoneally. Anesthesia was performed thirty minutes before pulp lesion. For plasma collection performed 14 h later, animals were re-anesthetized thirty minutes before the procedure [34]. All experimental procedures were conducted in accordance with the ethical guidelines established by the Institutional Bioethics Committee and the Official Mexican Norm (NOM-062-ZOO-1999). Animals were anesthetized with sodium pentobarbital prior to pulp exposure to minimize pain during the procedure. After the intervention, mice were closely monitored for signs of distress, including changes in posture, locomotion, grooming behavior, food and water intake, and response to external stimuli. Humane endpoints were predefined, and animals exhibiting severe or persistent signs of pain, distress, infection, or systemic deterioration were humanely euthanized with an anesthetic overdose, in accordance with institutional protocols. No unexpected morbidity or mortality was observed during the experimental period. Following recovery from anesthesia, animals were monitored for general wellbeing and behavior. All mice regained normal posture and mobility, and exhibited typical behaviors including ambulation, feeding, and grooming. No overt signs of distress or impaired activity were observed prior to sample collection.

#### 4.3.2. Anti-Inflammatory Treatment

The PGI_IBU_ group was treated with commercial ibuprofen, administered orally with a dose of 90 mg/Kg [34,206], thirty minutes before plasma collection at 14 h. The PGI_DEX_ group was treated with commercial dexamethasone sodium phosphate, administered intraperitoneally with a dose of 1 mg/Kg [34,182], ninety minutes before plasma collection at 14 h.

#### 4.3.3. Treatment with Resiniferatoxin

The control group RTX_HG_ and experimental PGI_RTX_ group were treated with resiniferatoxin (Sigma-Aldrich, 3050 Spruce St., Saint Louis, MO, USA, 63103), administered intraperitoneally [33,34] with a dose of 20 μg/Kg, ninety minutes before plasma collection at 14 h.

### 4.4. Plasma Collection

All the mice were administered 200 IU of heparin subcutaneously and fifteen minutes later the blood was obtained by the cardiac puncture method. For plasma collection, animals were re-anesthetized under deep anesthesia prior to cardiac puncture. Blood collection was performed as a terminal procedure, in accordance with institutional ethical guidelines and the Official Mexican Norm NOM-062-ZOO-1999. The blood was centrifuged for ten minutes at 10,000 rpm, and then the plasma was collected. After, aliquots of 60 μL were taken and deposited in Eppendorf tubes and stored at −80 °C until use.

### 4.5. Quantification of PGE_2_ and TNF-α in Plasma

Plasma samples from the HC_G_ group were collected at the same experimental time point as those from the RTX_HG_ group; the only difference between groups was the administration of resiniferatoxin to the RTX_HG_ group 90 min prior to sampling, then the concentrations of PGE_2_ and TNF-α in plasma were determined. In the PG_I_, group, the plasma was collected at 14 h after that pulp–gingival lesion, then the concentrations of PGE_2_ and TNF-α were determined. Whereas, in the PGI_IBU_, PGI_DEX_, and PGI_RTX_ groups, the plasma was collected at 14 h after pulp–gingival lesion, and then the concentrations of PGE_2_ and TNF-α were determined. Plasma levels of PGE_2_ were determined by ELISA using Mouse Prostaglandin E_2_ (PGE_2_) ELISA Kit, MyBioSource, San Diego, CA, USA. Plasma levels of TNF-α were determined by ELISA using Mouse TNF-alpha DuoSet^®^ ELISA, R&D Systems, Inc. a Bio-Techne Brand USA (Minneapolis, MN, USA). Plasma samples were analyzed in duplicate and diluted 1:2 when necessary. Standard curves were generated for each assay by serial dilutions of the provided standards, covering a concentration range of 7.8–500 pg/mL for PGE_2_, and 31.3–2000 pg/mL for TNF-α. Absorbance was measured using a microplate reader (450 nm), and cytokine concentrations were calculated based on the corresponding standard curves (Figure 1b, in Section 2 of results).

### 4.6. Histopathological Analysis

Although pulp tissue samples were obtained during tissue processing, the histopathological and quantitative analyses in this study focused exclusively on gingival tissue to assess periodontal inflammation secondary to pulp damage. This was because our group had already reported inflammatory changes in pulp tissue following mechanical damage and resiniferatoxin treatment [34], and therefore these were not included in the present analysis. For histopathological analysis of the gingival tissue, attached gingiva was removed from the first upper molars, fixed in 4% paraformaldehyde (PFA) in PBS at 4 °C for 24 h, and embedded in paraffin wax. Paraffin-embedded gingival tissues were sectioned at a thickness of 4–5 μm using a rotary microtome. Sections were obtained in a bucco–lingual orientation to ensure consistent anatomical evaluation. For each specimen, three non-consecutive levels were analyzed, with multiple sections examined per level to assess tissue architecture and inflammatory infiltrate. Subsequently, sections were cut and stained with H&E on a glass microscope slide [34].

Gingival tissue integrity and damage were evaluated based on the following histopathological criteria: (1) location of inflammatory cells (absent, restricted to the exposed site, limited to the gingival epithelium, or extending throughout the gingival connective tissue); (2) intensity of the inflammatory infiltrate (baseline: 0–20 inflammatory cells; mild: 21–40 inflammatory cells; moderate: 41–80 inflammatory cells; severe: over 80 inflammatory cells); and (3) inflammatory edema (absent and present). The inflammatory cells infiltrated into the gingival tissue were counted in 10 randomly selected microscopic fields per sample, analyzed in triplicate using a 40× objective, covering a total analyzed area of approximately 1.0 mm^2^. The results were expressed as the number of infiltrated inflammatory cells II_C_/TAU (Tissue Area Unit: 1 mm^2^). The histopathological criteria were evaluated under optical light microscope (Carl Zeiss Primo Star microscope Carl Zeiss Microscopy GmgH 37081 Gottingen, Germany, model 3708) with objectives 10× and 40× [34,85]. Histological scoring was performed in a blinded manner by a trained observer using predefined criteria.

### 4.7. Statistical Analysis

Results are presented as mean ± standard deviation (SD). To minimize Type I errors (false positives), a predefined significance level (* *p* < 0.05) was applied, and comparisons between groups were performed using a one-way ANOVA, suitable for controlling for overall variance across multiple groups. Regarding Type II errors (false negatives), the use of standardized experimental conditions, homogeneous animal populations, and effect sizes consistent with previous studies increased the likelihood of detecting biologically relevant differences, despite the limited sample size. When significant differences were detected, Tukey’s post hoc test was applied for multiple comparisons. Normality was assessed using the Shapiro–Wilk test, and homogeneity of variances was evaluated using Levene’s test. All datasets satisfied the assumptions of normality and homogeneity of variance required for parametric analysis. Statistical analyses were performed in GraphPad PRISM for Mac version 10 (GraphPad Software, San Diego, CA, USA).

## 5. Conclusions

In this study, systemic levels of proinflammatory mediators, including PGE_2_ and TNF-α, were detected during gingival inflammation following mechanical pulp damage in a murine experimental model. In addition, resiniferatoxin exhibited an anti-inflammatory effect, evidenced by significant reductions in plasma PGE_2_ and TNF-α levels, as well as decreased inflammatory cell infiltration in gingival tissue, with effects comparable to those observed with ibuprofen and dexamethasone. Within the constraints of this controlled preclinical model and the acute time window evaluated, these findings suggest that resiniferatoxin represents a promising experimental anti-inflammatory approach for modulating gingival inflammation secondary to pulp injury, warranting further investigation in complementary models and temporal settings.

## Figures and Tables

**Table 1:** Evidence on the effects of ibuprofen on PGE_2_ and TNF-α in pulpal and periodontal tissues.

Tissue/Model	Study Design	Inflammatory Mediator	Key Findings	Ref.
Inflamed oral tissues	Experimental Clinical	PGE_2_	Ibuprofen significantly reduces PGE_2_ levels in inflamed oral tissues.	[118,119,120]
Dental pulp	Experimental	PGE_2_	Ibuprofen inhibits arachidonic acid metabolism in pulp tissue.	[121]
Dental pulp (Exposed rats)	Experimental	PGE_2_	PGE_2_ levels were significantly increased in untreated rats and markedly reduced after ibuprofen treatment.	[122]
Dental pulp (Irreversible pulpitis)	Clinical	PGE_2_	Preoperative ibuprofen significantly decreased PGE_2_ levels in pulpal blood samples compared with untreated patients.	[118]
Inflamed gingival tissue	In vitro	PGE_2_	Ibuprofen significantly inhibited PGE_2_ production in homogenates of inflamed human gingival tissue.	[123]
Inflamed periodontal tissue	In vitro	PGE_2_	Ibuprofen inhibited the in vitro conversion of arachidonic acid to PGE_2_, showing moderate potency as a competitive COX inhibitor.	[124]
Moderate/advanced periodontitis	Randomized clinical trial	immunoreactive PGE_2_	Ibuprofen reduced tissue immunoreactive PGE_2_ levels by >95% and significantly decreased postoperative pain.	[125]
Natural periodontitis (Beagle dogs)	Experimental	PGE_2_ gingival crevicular fluid	PGE_2_ levels increased during disease progression and were significantly reduced with ibuprofen treatment.	[126]
Endo-periodontal lesions/severe chronic periodontitis	Clinical	TNF-α	TNF-α levels increased significantly, with no significant reduction after ibuprofen treatment in some studies.	[120,127,128]
Dental pulp (irreversible pulpitis)	Clinical	TNF-α	Ibuprofen significantly decreased TNF-α levels in pulpal blood samples.	[118]
Gingival epithelial cells	In vitro	TNF-α	Ibuprofen significantly downregulated TNF-α release in *P. gingivalis*-LPS-stimulated cells.	[129]
Pulp inflammation	Experimental	PGE_2_ TNF-α	Ibuprofen significantly decreased (*p* < 0.05) plasma levels of both mediators.	[34]
Molecular mechanisms	Experimental	TNF-α NF-κB	Ibuprofen attenuates TNF-α levels and downregulates NF-κB, suggesting immunomodulatory effects beyond COX inhibition	[130,131,132,133]

**Table 2:** Evidence on the effects of dexamethasone on pulp and periodontal inflammation.

Tissue/Model	Study Design	Inflammatory Mediator	Main Findings	Ref.
Pulp diseases	Clinical	Endodontic pain	Dexamethasone has been extensively evaluated for endodontic pain control, particularly in inferior alveolar nerve block.	[139,140,141,142,143,144]
Exposed dental pulp	Experimental (Rats)	TNF-α inflammation	Dexamethasone-loaded nanohydroxyapatite microspheres promoted dentin mineralization, inhibited TNF-α expression, reduced inflammation progression, and promoted reparative dentin formation.	[145]
Pulp inflammation	Experimental (Mouse)	PGE_2_ TNF-α	Dexamethasone significantly decreased (* *p* < 0.05) plasma levels of PGE_2_ and TNF-α at 14 and 18 h after pulp damage.	[34]
Periodontal disease	Clinical	Postoperative pain	Dexamethasone treatment showed a preventive effect on postoperative periodontal pain.	[146]
Gingival tissue and periodontal ligament	In vitro	mPGES-1 COX-2 PGE_2_ TNF-α	Dexamethasone inhibited mPGES-1 and COX-2 expression and reduced PGE_2_ and TNF-α production in stimulated human gingival fibroblasts and periodontal ligament cells.	[153,154,155,156,160,161,162]
Acute periodontitis	Experimental (rat)	TNF-α	Dexamethasone significantly inhibited the increase in TNF-α associated with acute periodontitis.	[163]
Experimental periodontitis	Experimental (rat)	Inflammation alveolar bone	Dexamethasone reduced inflammation and alveolar bone loss but also altered mineral density and increased bone porosity.	[164,165,166,167]

## Data Availability

The original contributions presented in this study are included in the article. Further inquiries can be directed to the corresponding authors.
